# Targeting immune checkpoints in unresectable metastatic cutaneous melanoma: a systematic review and meta‐analysis of anti‐CTLA‐4 and anti‐PD‐1 agents trials

**DOI:** 10.1002/cam4.732

**Published:** 2016-05-11

**Authors:** Seongseok Yun, Nicole D. Vincelette, Myke R. Green, Andrea E. Wahner Hendrickson, Ivo Abraham

**Affiliations:** ^1^ Department of MedicineUniversity of ArizonaTucsonArizona85721; ^2^ Hematology and OncologyUniversity of ArizonaTucsonArizona85721; ^3^ Molecular Pharmacology and Experimental TherapeuticsMayo ClinicRochesterMinnesota55905; ^4^ Medical OncologyMayo ClinicRochesterMinnesota55905; ^5^ Center for Health Outcomes and PharmacoEconomic ResearchUniversity of ArizonaTucsonArizona85721; ^6^ Arizona Cancer CenterUniversity of ArizonaTucsonArizona85721

**Keywords:** CTLA‐4, Ipilimumab, Lambrolizumab, metastatic melanoma, nivolumab, PD‐1, pembrolizumab, tremelimumab

## Abstract

Anti‐cytotoxic T lymphocyte‐associated antigen‐4 (CTLA‐4) and anti‐programmed cell death‐1 (PD‐1) inhibitors have been shown to significantly improve survival in patients with metastatic cutaneous melanoma. However, there was some heterogeneity as well as some variation in the degree of benefit across studies. We reviewed randomized trials and performed a meta‐analysis to determine the efficacy and safety of immune checkpoint inhibitors in comparison with conventional regimens. Eligible studies were limited to randomized controlled trials comparing anti‐CTLA‐4 or anti‐PD‐1 inhibitors to chemotherapy or vaccination treatment in adult patients with unresectable cutaneous metastatic melanoma. Progression‐free survival (PFS) rate at 6 months was 28.5% versus 17.7% (RR: 0.84, 95% CI: 0.76–0.93), overall survival (OS) rate at 1 year was 51.2% versus 38.8% (RR: 0.72, 95% CI: 0.59–0.88), and overall response rate (ORR) at 6 months was 29.6% versus 17.7% (RR: 0.85, 95% CI: 0.76–0.95) favoring immune check point inhibitors over chemotherapies or vaccination. Immune check point inhibitors were associated with more frequent immune‐related adverse events at 13.7% versus 2.4% of treated patients (RR: 6.74, 95% CI: 4.65–9.75). Subgroup analyses demonstrated significant PFS (RR: 0.92 vs. 0.74, *P *<* *0.00001) and ORR (RR: 0.95 vs. 0.76, *P *=* *0.0004) improvement with anti‐PD‐1 treatment compared to anti‐CTLA‐4 when each of them was compared to control treatments. Collectively, these results demonstrate that immune checkpoint inhibitors have superior outcomes compared to conventional chemotherapies or vaccination, and support the results of recent randomized trials that showed superior outcomes with anti‐PD‐1 agents over ipilimumab in unresectable metastatic cutaneous melanoma patients.

## Introduction

Metastatic cutaneous melanoma had a poor prognosis with a 2‐year survival rate of <20% with conventional chemotherapies. Targeted therapies such as v‐raf murine sarcoma viral oncogene homolog B1 (BRAF) and MEK inhibitors have shown significant survival advantage in BRAF‐mutant melanoma 1, 2, 3, 4. However, 50–60% of patients with wild‐type BRAF require distinctive therapeutic approaches due to clinical resistance to these agents. Ipilimumab, an anti‐CTLA‐4 (cytotoxic T lymphocyte‐associated antigen‐4) human monoclonal antibody (IgG1) that blocks the T‐cell co‐inhibitory signal 5, 6 demonstrated significant survival benefit in metastatic melanoma patients regardless of BRAF mutation status, whereas tremelimumab, another anti‐CTLA‐4 IgG2 monoclonal antibody, failed to show such benefit 7. Peripheral tissues and tumor cells express PD‐L1, which neutralizes T‐cell antitumor immunity via PD‐1‐mediated co‐inhibitory signal 8. Accordingly, anti‐PD‐1 treatments have been shown to increase T‐cell antitumor activity through independent mechanisms from anti‐CTLA‐4 inhibitor treatment 9, 10. Recent randomized trials with nivolumab and pembrolizumab have demonstrated a survival advantage, including patients who progressed after antecedent ipilimumab treatment 11, 12, 13, 14. However, there was some heterogeneity and the degree of benefit seems to vary across studies. Therefore, we performed a systematic review and meta‐analysis to determine the efficacy and safety of immune checkpoint inhibitors as a category in comparison with conventional chemo‐ or vaccination treatments.

## Materials and Methods

### Study selection criteria

Eligible studies were (1) randomized controlled trials, (2) assessing patients with unresectable metastatic cutaneous melanoma, (3) treated with either immune check point inhibitors (ipilimumab, tremelimumab, nivolumab, pembrolizumab [previously known as lambrolizumab]) versus chemotherapy or vaccination (dacarbazine, carboplatin, temozolomide, paclitaxel, or gp100), and (4) reporting 6 months PFS and treatment response outcomes. Trials were used only once in the analysis using the most updated available data.

### Data sources

Literature search and review of relevant articles were limited to human studies. Key words included metastatic melanoma, CTLA‐4, PD‐1, ipilimumab, tremelimumab, nivolumab, pembrolizumab, and lambrolizumab (Table S1). Relevant studies were identified by searching PubMed, EMBASE, and Cochrane database of systematic review up to Sep 2015. A bibliography of identified articles and additional literatures from relevant references were further investigated manually to identify any relevant studies.

### Data extraction and assessment of bias risk

Two reviewers (S.Y. and N.D.V.) independently extracted data with a piloted extraction form and conducted the bias risk assessment using the Cochrane Collaboration tool (Table S3) 15. Any disagreement was resolved by consensus with a third author (M.R.G.). The following information was extracted from individual trial reports: publication year, inclusion/exclusion criteria, sample size, median age, American Joint Committee on Cancer (AJCC) Stage, BRAF mutation status, PD‐L1 positivity, number of prior systemic treatments, response to previous ipilimumab treatment, PFS, OS, ORR (defined as rate of complete remission or partial remission), adverse events, and mortality attributed to disease progression. Extracted from each study report were the number of patients treated with immune check point inhibitors or conventional treatments, number of events (death, treatment response, and treatment‐ or immune‐related adverse events), results from subgroup analyses, risk ratio (RR), odd ratio (OR), hazard ratio (HR), 95% CI, and *P* values. The primary outcome measures in this meta‐analysis were the 6‐month PFS rate and ORR from treatment. Secondary outcomes included the 1‐year OS rate from treatment and the grade 3/4 immune‐related adverse events rate.

### Statistical analysis

Statistical analysis was performed as described in a different meta‐analysis 16. Briefly, meta‐analysis calculations were performed using RevMan Version 5.3 (Copenhagen: The Nordic Cochrane Centre, 2014). We used the Cochran Q statistic to estimate statistical heterogeneity and the *I* ^2^ statistic to quantify inconsistency. The assumption of homogeneity was considered invalid if *P *<* *0.10. Treatment effects were calculated with a random effects model. The funnel plot method was applied to assess publication bias. A two‐sided *P *≤* *0.05 was considered statistically significant in the RR analysis. We performed an intention‐to‐treat analysis following allocated treatments for the overall response and survival outcomes, and per protocol analyses for the treatment‐related adverse events. Predefined criteria including experimental agent (anti‐CTLA‐4 vs. anti‐PD‐1), response to prior ipilimumab treatment (naïve vs. refractory), BRAF mutation status (wild‐type vs. V600E mutation), and PD‐L1 positivity (expression level >5% vs. ≤5%) were used for subgroup analyses to explore heterogeneity and to identify subgroups with differential benefit from the experimental agents (Table 2). The RR differences between subgroups were evaluated by meta‐regression models.

## Results

### Search results

**Figure 1:**
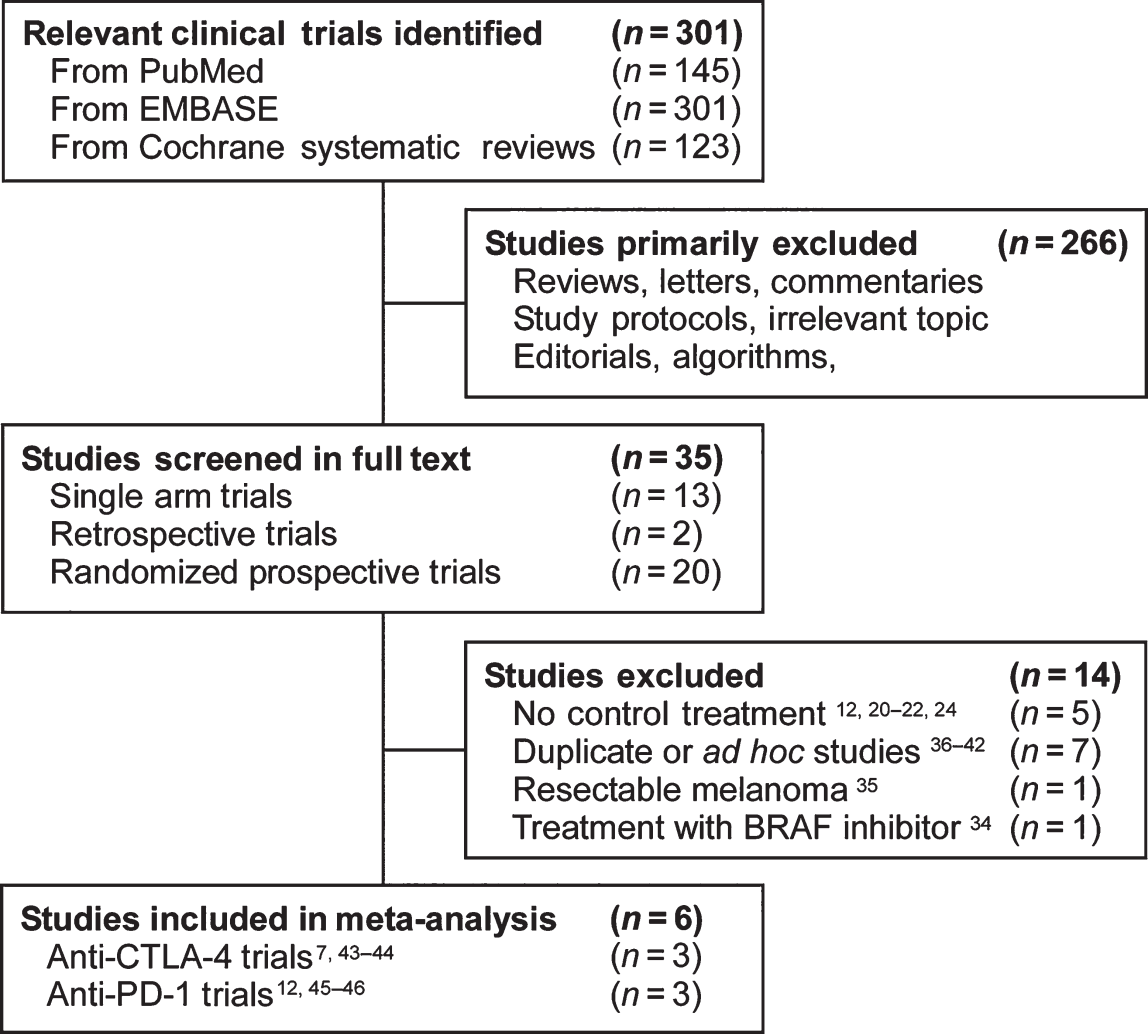
Trials selection process for the meta‐analysis.

Our initial literature search yielded a total 301 relevant abstracts (Figs 1 and S1**)**. Of these, 266 studies including commentaries, editorials, study protocols, and algorithm were excluded for being irrelevant based on abstract review. The remaining 35 studies were reviewed in full text. Of these, two retrospective studies 17, 18, 18 single arm or randomized studies without control treatment 11, 13, 14, 19, 20, 21, 22, 23, 24, 25, 26, 27, 28, 29, 30, 31, 32, 33, one study with sequential treatment with a BRAF inhibitor 34, and one study with resectable melanoma 35 were excluded from the meta‐analysis. Seven duplicate or ad hoc studies 36, 37, 38, 39, 40, 41, 42 were also excluded. Following verification of eligibility, a total of six phase II or III randomized controlled trials (three with anti‐CTLA‐4 7, 43, 44 and three with anti‐PD‐1 12, 45, 46 monoclonal antibodies) were selected for the meta‐analysis. The characteristics of these trials are summarized in Tables**** 1 and S2.

**Table 1:** Characteristics of trials included in the meta‐analysis

Study	Exp. drug	Median age (Range)		Pathology	Stage (%)^a^	No. of prior systemic Tx (%)	BRAF mutant (%)	PD‐L1 positivity (%)	ECOG (%)	Treatment		No. of Patients	
		Exp	Ctrl							Exp	Ctrl	Exp	Ctrl
Hodi et al. 44NCT00094653	Ipilimumab	56 (NR)	57 (NR)	HLA–A*0201‐positive Cutaneous Melanoma	M0, M1a, or M1b: 193 (29)M1c: 483 (71)	0: 0 (0)≥1: 676 (100)	NR	NR	0: 374 (55)1: 291 (43)2: 9 (1)3: 1 (0.1)	Ipilimumab (3 mg/kg) ± gp100	Gp100	540	136
Robert et al. 43NCT00324155	Ipilimumab	57.5 (NR)	56.4 (NR)	Cutaneous Melanoma	M0, M1a, or M1b: 220 (44)M1c: 282 (56)	0: 369 (73)≥1: 133 (27)	NR	NR	0: 356 (71)1: 146 (29)	Ipilimumab (10 mg/kg) + dacarbazine	Placebo + dacarbazine	250	252
Ribas et al. 7NCT00257205	Tremelimumab	56 (22–90)	57 (22–90)	CutaneousMelanoma	IIIc: 33 (5)M1a: 96 (15)M1b: 144 (22)M1c: 382 (58)	0: 655 (100)	NR	NR	0: 449 (69)1: 191 (29)	Tremelimumab (15 mg/kg)	Dacarbazine or temozolimide	327	328
Weber et al. 12NCT01721746	Nivolumab	59 (23–88)	62 (29–85)	Cutaneous Melanoma	III: 13 (3)IV: 392 (97)	0: 0 (0)1: 111 (27)2: 207 (51) ≥3: 87 (21)	89 (22)	259 (65)^b^	0: 246 (61)1: 158 (39)	Nivolumab (3 mg/kg)	Dacarbazine or paclitaxel	272	133
Robert et al. 45NCT01721772	Nivolumab	64 (18–86)	66 (26–87)	Cutaneous Melanoma	M0, M1a, or M1b: 163 (39)M1c: 266 (61)	0: 348 (83)≥1: 70 (17)	0 (0)	148 (35)^b^^,^ c	0: 269 (64)1: 144 (34)2: 4 (1)	Nivolumab (3 mg/kg)	Dacarbazine	210	208
Ribas et al. 46NCT01704287	Pembrolizumab	61 (15–89)	63 (27–87)	CutaneousMelanoma	M0: 4 (<1)M1a: 37 (7)M1b: 54 (10)M1c: 445 (82)	0: 1 (<1)1: 143 (26)2: 223 (41)≥3: 173 (32)	125 (23)^d^	NR	0: 295 (55)1: 163 (30)	Pembrolizumab (2 mg/kg or 10 mg/kg)	Carboplatin + paclitaxel, carboplatin, paclitaxel, dacarbazine, or temozolomide	361	179

a The metastasis stage was defined according to the tumor‐node‐metastasis system of the American Joint Committee on Cancer and the International Union against Cancer 47.

b PD‐L1 positivity was defined as at least 5% of tumor cells exhibiting cell surface PD‐L1 staining of any intensity in a section containing at least 100 evaluable cells. PD‐L1 expression was assessed in a central laboratory with an automated Bristol‐Myers Squibb/Dako immunohistochemistry assay using rabbit monoclonal antihuman PD‐L1 antibody (clone 28–8) 21. Antibody specificity was tested with western blot.

c PD‐L1 negative and indeterminate groups were calculated together for subgroup analysis in this trial.

d BRAF mutation indicates BRAF^V600^.

HLA, human leukocyte antigen; BRAF, v‐raf murine sarcoma viral oncogene homolog B1; PD‐1, programmed cell death‐1; PD‐L1, PD‐ligand 1; ECOG, Eastern Cooperative Oncology Group; Exp, experimental treatment group; Ctrl, control treatment group; Tx, treatment, NR, not reported.

### Patients

All trials included patients with histologically confirmed metastatic cutaneous melanoma. The range of median age of patients across these studies was 56–66 years. A total of 3196 patients were included in the meta‐analysis. Of these, 1960 were treated with either ipilimumab (*n* = 790), tremelimumab (*n* = 327), nivolumab (*n* = 482), or pembrolizumab (*n* = 361) and 1236 with chemotherapies (dacarbazine, carboplatin, temozolomide, or paclitaxel) (*n* = 1100) or gp100 (*n* = 136) (Table**** 1). Metastasis stage was defined according to AJCC tumor‐node‐metastasis system 47. There were 1858 patients classified as M1c and a total of 911 patients were either M0, M1a, or M1b (Table**** 1). BRAF mutation status and PD‐L1 positivity were reported in three studies with anti‐PD‐1 treatment 12, 45, 46. The numbers of patients with BRAF mutation, PD‐L1 positivity, no prior systemic treatment, and ipilimumab refractory disease were 89, 407, 1373, and 945, respectively (Table S2). In total, 48% 46, 3.8% 45, and 16.0% 7 of patients in the control groups of three studies were treated with pembrolizumab, nivolumab, and ipilimumab, respectively, while the remaining trials did not have crossover options.

### RR of survival and treatment response rate

**Figure 2:**
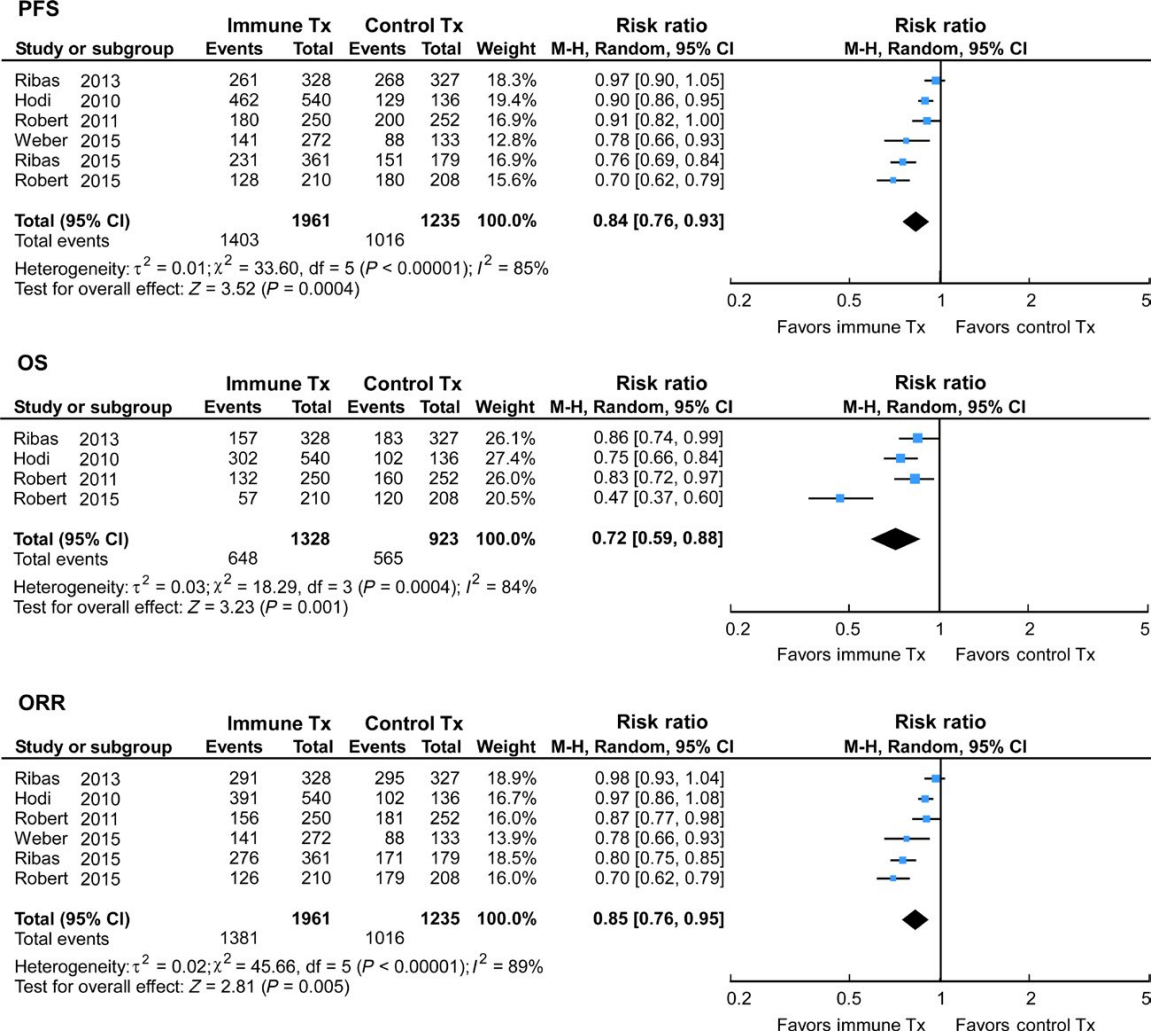
Survival and treatment response outcomes from available data. Forest plots of risk ratio for PFS (at 6 months), OS (at 1 year), and ORR (at 6 months) from all available data. The size of the data markers (square) corresponds to the weight of the study in the meta‐analysis. The effects of interventions are calculated with the random effects model.

Immune check point inhibitors were associated with higher 6‐month PFS rate of 28.5% versus 17.7% (RR: 0.84, 95% CI: 0.76–0.93, *P *=* *0.0004), 1‐year OS rate of 51.2% versus 38.8% (RR: 0.72, 95% CI: 0.59–0.88, *P *=* *0.001), and higher ORR of 29.6% versus 17.7% (RR: 0.85, 95% CI: 0.76–0.95, *P *=* *0.005) (Fig. 2). Grade 3/4 immune‐related adverse events were more frequently associated with immune check point inhibitors at 13.7% versus 2.4% (RR: 6.74, 95% CI: 4.65–9.75, *P *<* *0.0001) based on per protocol analysis. There was significant heterogeneity in PFS (*I* ^2 ^= 85%, *P *<* *0.00001), OS (*I* ^2 ^= 84%, *P *=* *0.0004), and ORR (*I* ^2 ^= 89%, *P *<* *0.00001) analyses, but not in immune‐related adverse events (*I* ^2 ^= 0%, *P *=* *0.48) across studies (Figs. 2 and 2S).

### Subgroup analyses

Both anti‐CTLA‐4 and anti‐PD‐1 inhibitor treatments were associated with higher PFS rates when each treatment was compared to control, however, with a significant subgroup difference favoring nivolumab or pembrolizumab over ipilimumab or tremelimumab treatments (RR: 0.92 vs. 0.74, *P *<* *0.00001) (Table 2). The *I* ^2^ statistics for the anti‐CTLA and anti‐PD‐1 subgroups were 31% and 0%, explaining the heterogeneity observed in the PFS analysis (Fig. S3A). In trials investigating anti‐PD‐1 treatment, PFS in patients with ipilimumab refractory disease was not different from that of ipilimumab‐naïve patients (RR: 0.77 vs. 0.70, *P *=* *0.27) (Fig. S3B). Similarly, the ORR for anti‐PD‐1 treatment was significantly higher than the ORR for anti‐CTLA‐4 treatment (RR: 0.95 vs. 0.76, *P *=* *0.0004) (Table 3, Fig. S4A and B). In patients treated with nivolumab or pembrolizumab, PD‐L1‐positive and ipilimumab‐naïve patients had better ORR compared to PD‐L1‐negative (RR: 0.57 vs. 0.84, *P *=* *0.001) and ipilimumab‐refractory patients (RR: 0.70 vs. 0.80, *P *=* *0.05) (Tables 2 and 3). BRAF mutation status did not have a statistically significant prognostic impact on ORR (RR: 0.84 vs. 0.85, *P = *0.97) (Tables 2 and 3, Figs. S3 and S4).

**Table 2:** Subgroup analysis of PFS rate from available data

Subgroup		No. of Studies	RR (95% CI)^‡^	Weight (%)	Heterogeneity within subgroup	
Criteria	Characteristics				*I* ^2^ (%)	*P* value
Experimental drug	Anti‐CTLA‐4	3	0.92 (0.88, 0.97)	54.7	31	0.24
	Anti‐PD‐1	3	0.74 (0.69, 0.80)	45.3	0	0.52
	Subgroup difference	*P *<* *0.00001^b^				
Ipilimumab naïve versus refractory disease^a^	Ipilimumab naïve	1	0.70 (0.62, 0.79)	33.5	NA	NA
	Ipilimumab refractory	2	0.77 (0.70, 0.83)	66.5	0	0.52
	Subgroup difference	*P *=* *0.27				

a Studies with nivolumab or pembrolizumab were used for the subgroup analyses.

b Statistically significant.

CTLA‐4, cytotoxic T lymphocyte‐associated protein‐4; PD‐1, programmed cell death‐1; RR, risk ratio.

**Table 3:** Subgroup analysis of ORR from available data

Subgroup		No. of Studies	RR (95% CI)	Weight (%)	Heterogeneity within subgroup	
Criteria	Characteristics				*I* ^2^ (%)	*P* value
Experimental drug	Anti‐CTLA‐4	3	0.95 (0.88, 1.02)	51.6	50	0.13
	Anti‐PD‐1	3	0.76 (0.69, 0.84)	48.4	54	0.12
	Subgroup difference	*P *<* *0.00001^c^				
Ipilimumab naïve versus refractory disease^a^	Ipilimumab naïve	1	0.70 (0.62, 0.79)	30.5	NA	NA
	Ipilimumab refractory	2	0.80 (0.75, 0.85)	69.5	0	0.78
	Subgroup Difference	*P *=* *0.05^c^				
BRAF mutation^a^	BRAF wild‐type	2	0.84 (0.68, 1.03)	81.4	76	0.04
	BRAF mutant	1	0.85 (0.64, 1.12)	18.6	NA	NA
	Subgroup Difference	*P *=* *0.97				
PD‐L1 status^a^	PD‐L1 positive^b^	2	0.57 (0.48, 0.69)	45.4	0	0.38
	PD‐L1 negative	2	0.84 (0.73, 0.96)	54.6	29	0.24
	Subgroup Difference	*P *=* *0.001^c^				

a Data from nivolumab and pembrolizumab trials were used for these subgroup analyses.

b PD‐L1 positivity was defined as at least 5% of tumor cells exhibiting cell surface PD‐L1 staining of any intensity in a section containing at least 100 evaluable cells. Patients with indeterminate PD‐L1 expression level were included into PD‐L1‐negative group for the subgroup analysis in study performed by Robert *et al* 45.

c Statistically significant.

CTLA‐4, cytotoxic T lymphocyte‐associated protein‐4; PD‐1, programmed cell death‐1; PD‐L1, PD‐ligand 1; RR, risk ratio; BRAF, v‐raf murine sarcoma viral oncogene homolog B1).

### Bias analysis

Four trials were double‐blinded and two were open‐label studies 7, 12. Random sequence generation and allocation concealment were performed adequately in all studies. The adequacy of blinding was judged by whether treatment response was evaluated by a third person who did not know the treatment group of the patients. Four studies 12, 43, 45, 46 performed blinded assessments, but blinding was unclear in two studies 7, 44 (Table S3). The baseline demographic characteristics were balanced in all trials (Tables**** 1 and S2). Potential sources of bias are described in Table S3. PFS and ORR analyses showed heterogeneity, largely attributable to the experimental agent used (anti‐CTLA‐4 vs. anti‐PD‐1) and the significant subgroup difference observed, but these PFS and ORR subgroup analyses also evidenced intra‐subgroup homogeneity (Tables 2 and 3). The observed funnel plot asymmetry can also be explained as a function of experimental agent used (Fig. S1).

## Discussion

Although the benefit of immune checkpoint inhibitors as a class has been observed consistently in previous randomized trials, some of the agents failed to show benefit 7 and the efficacy of immune checkpoint inhibitors seems to be variable. Meta‐analysis, in general, obtains a quantitative synthesis from studies with similar design to estimate the overall effect of interventions and to improve the precision of estimates of treatment effects 48, 49. Therefore, we performed a meta‐analysis comparing the outcomes of immune checkpoint inhibitors as a category to conventional chemotherapies or vaccination in patients with unresectable metastatic cutaneous melanoma, with a focus on subgroup analyses to explain the heterogeneity across studies and to identify subgroups that are associated with better clinical outcomes.

The pooled analyses revealed statistically significant PFS, OS, and ORR benefits with immune check point inhibitors (Fig. 2), suggesting the superiority of immune checkpoint inhibitors over conventional regimens. Both anti‐CTLA‐4 and anti‐PD‐1 treatments were associated with clinical benefit in our meta‐analysis; however, an indirect comparison of these two agents showed superior PFS and ORR in anti‐PD‐1 compared to anti‐CTLA‐4 treatment (Tables 2 and 3). This result is consistent with data from two recent randomized trials that were published while our study was ongoing. The KEYNOTE‐006 trial showed higher PFS, OS, and ORR with two different treatment schedules of pembrolizumab treatment (10 mg/kg every 2 weeks and 3 weeks) compared to ipilimumab 50. The CheckMate 067 trial revealed PFS and ORR improvement with nivolumab (3 mg/kg every 2 weeks) compared to ipilimumab 51. Ipilimumab used to be the standard first‐line treatment for advanced metastatic melanoma based on results from phase II and III trials 22, 23, 43, 44, however, the prevailing guidelines 52 recommend either anti‐PD‐1 monotherapy or nivolumab and ipilimumab combination therapy as the standard first‐line treatment in unresectable metastatic melanoma based on these two randomized trials 50, 51.

Daud et al. showed nominally higher ORRs (38% vs. 29%), 1‐year PFS rates (36% vs. 32%), and 1‐year OS rates (71% vs. 63%) in the ipilimumab naïve versus treated patients in their pooled analysis of 655 patients who were treated with pembrolizumab 53. Similarly, in our subgroup analysis, the ORR of nivolumab treatment in ipilimumab‐refractory patients was lower compared to ipilimumab‐naïve patients, although ORRs in both groups were still better than those in control treatments (Fig. S4B). Collectively, these results suggest that there is a certain patient population that may selectively respond to anti‐PD‐1 treatment and benefit from combination treatment of anti‐CTLA‐4 with anti‐PD‐1 agents. Similar to our results, the CheckMate 067 trial demonstrated ipilimumab and nivolumab combination treatment to have better ORR compared to nivolumab monotherapy, especially in PD‐L1‐positive patients 51, although this study was not designed for statistical comparison of combination treatment versus nivolumab monotherapy. Future randomized trials comparing anti‐CTLA‐4 and anti‐PD‐1 combination versus anti‐PD‐1 monotherapy in ipilimumab‐naïve and ‐refractory patients would provide valuable information to clarify the optimal first‐line treatment.

In prior study of nivolumab, PD‐L1 positivity, defined as more than 5% by immunohistochemistry staining, was associated with better response, although the association disappeared when a cutoff value of 1% was used as the positivity criteria 13. In other studies that used the 5% threshold, PD‐L1‐positive groups had significantly higher ORR in comparison with PD‐L1‐negative groups 20, 54. Similarly, in our meta‐analysis, the subgroup with PD‐L1 positivity (more than 5%) had a better treatment response to anti‐PD‐1 agents compared to the PD‐L1‐negative subgroup (Fig. S4D). However, the PD‐L1‐negative group still had significant ORR improvement in comparison with control treatments. This indicates that PD‐L1 positivity should not be used to select patients for anti‐PD‐1 treatment. Our results are supported by the recent CheckMate 069 trial that showed no ORR difference between PD‐L1 positive versus negative groups when patients received nivolumab and ipilimumab combination therapy 55. On the other hand, the CheckMate 067 trial showed a nominally higher ORR in the PD‐L1‐positive group over the PD‐L1‐negative group when these patients were treated with ipilimumab and nivolumab combination therapy or with nivolumab monotherapy 51. PD‐L1 expression levels were shown to be variable in different metastatic lesions in the same patient and anti‐PD‐1 treatment response also seemed to be affected by tumor mutational load and preexisting intratumor CD8 +  T‐cells based on preclinical studies 56, 57, 58. Collectively, the prognostic impact of PD‐L1 status needs further investigation.

Previous studies have suggested that BRAF mutation status does not affect the efficacy of checkpoint inhibitors 21, 59, 60. In our subgroup analysis, the ORR of patients with BRAF WT did not differ from that of BRAF mutation (Fig. S4C). Larkin et al. performed a pooled analysis from four studies including phase I and III trials to compare the clinical outcomes between patients with and without BRAF mutation who were treated with nivolumab 61. Although, this study included data from nonrandomized trials and was retrospectively analyzed, the ORR in BRAF WT versus mutant patients was 34.6% versus 29.7% with no statistically significant difference, which is consistent with our subgroup analysis.

We recognize several limitations of the current meta‐analysis. First, there was significant statistical heterogeneity in PFS and ORR analyses (*I* ^2 ^= 85%, *P *<* *0.00001 and *I* ^2 ^= 89%, *P *=* *0.005). However, this was predicted since data from two different classes of immune check point inhibitors were analyzed together. Accordingly, the primary source of the heterogeneity was from experimental agents as evidenced also by the intra‐subgroup homogeneity (*I* ^2 ^= 31%, *P *=* *0.24 and *I* ^2 ^= 0%, *P *=* *0.52 in PFS analyses of anti‐CTLA‐4 and anti‐PD‐1 treatment, respectively) (Fig. S3A). In recent studies, an increase in T‐cell receptor (TCR) repertoire was observed in patients treated with anti‐CTLA‐4 agent 62, and anti‐PD‐1 agents were shown to induce intratumoral CD8 +  T‐cells proliferation in patients who responded to therapy 57. Also, combination treatment of anti‐CTLA‐4 and anti‐PD‐1 showed distinct immunologic effects in vivo compared to single agent 63. Collectively, the biological differences in mechanisms of action between these two agents may lead to the heterogeneity observed in the clinical outcomes. There was some residual heterogeneity in the anti‐CTLA‐4 subgroup (*I* ^2 ^= 31%), mainly secondary to the tremelimumab trial 7 that showed no significant benefit from the experimental agent. In this study, patients with lactate dehydrogenase (LDH) higher than two times upper limit were excluded and 16% of patients in the control group were treated with ipilimumab, which may have masked the benefit of tremelimumab treatment. Second, the emergence of irRECIST criteria in 2009 for adjudicating immune‐related treatment response and the interchangeable use of both RECIST and irRECIST may have influenced the outcomes reported in the studies included in our analyses. The primary difference between these two sets of response criteria is the rate of alternative forms of response captured by irRECIST, but coded as progressive disease (PD) by RECIST criteria. The discrepancy in treatment response, estimated to be ~10% for ipilimumab and ~5–10% for anti‐PD‐1 agents, warrant caution in the interpretation of results 64, 65. Third, the PD‐L1 negative subgroup in one study 50 included patients with an indeterminate PD‐L1 level. Fourth, the sample size in each subgroup was relatively small, rendering the prognostic impact of PD‐L1 positivity less conclusive. Fifth, two ipilimumab 43, 44 and one tremelimumab 7 trials did not report BRAF mutation status. Lastly, median survivals of experimental groups in two studies 12, 42 were not reached yet and 1‐year OS rates were not reported. Therefore, the OS result should be interpreted with due caution and needs longer follow‐up.

Several questions remain to be answered. Immune checkpoint inhibitors are associated with significant risk of immune‐related adverse events in a range of 10–40% 22, 24, 43, 66, 67, and our meta‐analysis also demonstrated statistically higher grade 3/4 immune‐mediated adverse events rate in the experimental group (Fig. S2). Recently, nivolumab and ipilimumab combination treatment (which may have better efficacy than monotherapy) was shown to have even higher drug‐related adverse events than ipilimumab monotherapy, which became the most common reason for discontinuation of treatment 51, 55. Most of the immune‐related grade 3/4 adverse events can be effectively managed with either systemic steroid or infliximab therapy, but prophylaxis with budesonide failed to prevent immune‐related adverse events or improve survival outcomes 24. A recent trial showed that sargramostim combined with ipilimumab (10 mg/kg) not only enhanced efficacy but also reduced adverse events, specifically gastrointestinal and pulmonary adverse events, the former of which is the leading cause of treatment disruption and discontinuation 26. Further study is needed to maximize the benefit of immune check point targeting agents by reducing drug‐related adverse events. Second, BRAF inhibitor as a single agent and in combination with a MEK inhibitor was shown to improve survival in patients with BRAF mutation 1, 2, 3, 4, and immune check point inhibitors are effective regardless of BRAF mutation status 21, 59, 60. Therefore, the optimal sequence for treatment, especially in patients with BRAF mutation, needs further investigation. Third, the optimal dose and schedule of immune check point inhibitors remain to be determined. Lastly, the prognostic impact of PD‐L1 expression level as well as independent prognostic factors to anti‐CTLA‐4 and anti‐PD‐1 treatments need further investigation for better patient selection and improved clinical outcomes.

## Conclusion

In a meta‐analysis of randomized controlled trials with unresectable cutaneous metastatic melanoma patients, agents targeting immune checkpoints were associated with better PFS, OS, and ORR compared to conventional treatments. Subgroup analyses showed that survival benefit was significantly higher with anti‐PD‐1 treatment regardless of previous response to ipilimumab treatment, suggesting that nivolumab or pembrolizumab is a better choice as the first‐line treatment. Our meta‐analysis also indicates that there is a need for future study to assess the prognostic values of PD‐L1 expression level and optimal sequential treatments for better clinical outcome.

## Conflict of Interest

The authors declare that there is no conflict of interest.

## Supporting information


**Figure S1.** Funnel Plot.
**Figure S2.** Grade 3/4 immune‐related adverse events.
**Figure S3.** Subgroup analysis of 6‐month PFS rate from available data.
**Figure S4.** Subgroup analysis of 6‐month ORR from available data.
**Table S1.** Search detail in PubMed, EMBASE, and Cochrane database.
**Table S2.** Additional characteristics of included trials.
**Table S3**. Risk of bias assessment of studies according to Cochrane risk bias assessment tool 8.
