# Supplementary material for: Targeting immune checkpoints in unresectable metastatic cutaneous melanoma: a systematic review and meta‐analysis of anti‐CTLA‐4 and anti‐PD‐1 agents trials
Source: Cancer Med. 2016 May 11;5(7):1481–91. doi: 10.1002/cam4.732 (PMC4867662; doi:10.1002/cam4.732)
Supplement: Supplementary file 1 — Figure S1. Funnel Plot. Figure S2. Grade 3/4 immune‐related adverse events. Figure S3. Subgroup analysis of 6‐month PFS rate from available data. Figure S4. Subgroup analysis of 6‐month ORR from available data. Table S1. Search detail in PubMed, EMBASE, and Cochrane database. Table S2. Additional characteristics of included trials. Table S3. Risk of bias assessment of studies according to Cochrane risk bias assessment tool 8. [file CAM4-5-1481-s001.doc]

**Supplementary Materials to**

**Targeting Immune Checkpoints in Unresectable Metastatic Cutaneous Melanoma: a Systematic Review and Meta-analysis of anti-CTLA-4 and anti-PD-1 Agents Trials**

Seongseok Yun MD, PhD, Nicole D. Vincelette BS, Myke R. Green PharmD, BCOP, Andrea E. Wahner Hendrickson MD, and Ivo Abraham PhD

**Supplemental figure 1: Funnel Plot**

**
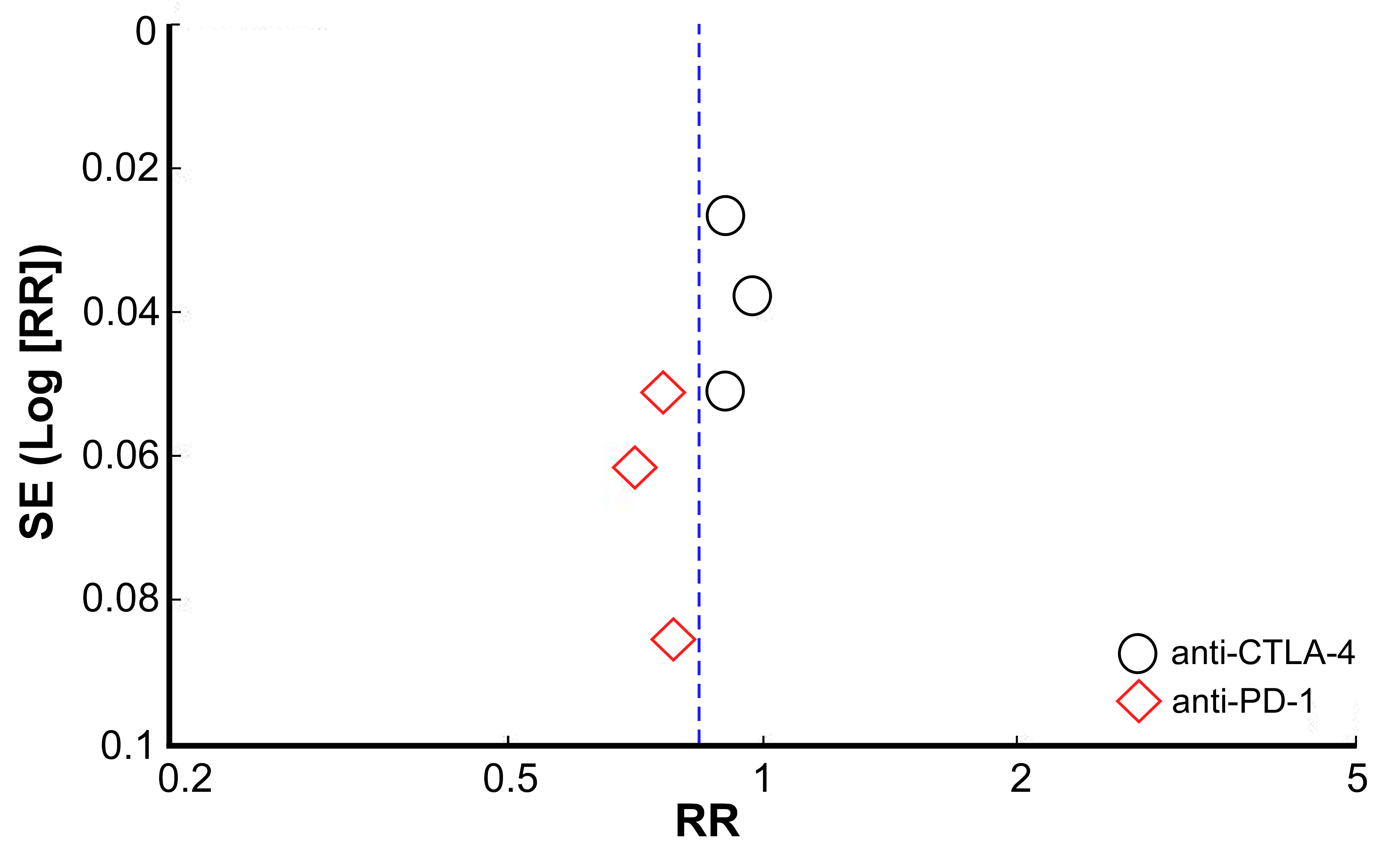
**

Funnel plot to evaluate the evidence of publication bias. Circle and diamond stand for trials of anti-CTLA-4 and anti-PD-1 treatments, respectively.

**Supplementary Figure 2: Grade 3/4 Immune-Related Adverse Events**

**
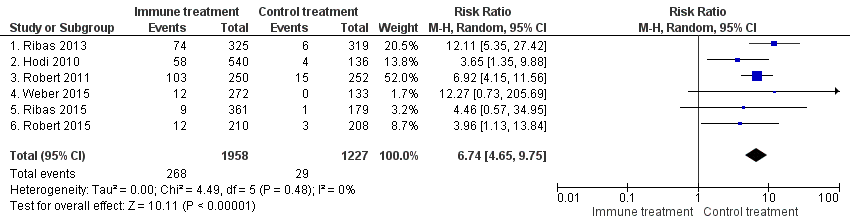
**

Forest plots of risk ratio for grade 3/4 immune-related adverse events. The size of the data markers (square) corresponds to the weight of the study in the meta-analysis. The effects of interventions are calculated with the random effects model.

**Supplementary Figure 3: Subgroup Analysis of 6 months PFS Rate from Available Data**

**S3A: anti-CTLA-4 vs. anti-PD-1 treatment**

**
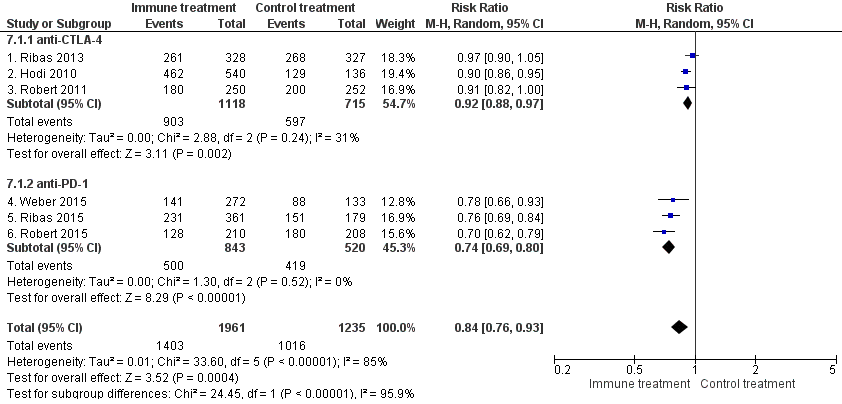
**

**S3B: ipilimumab naïve vs. ipilimumab refractory**

**
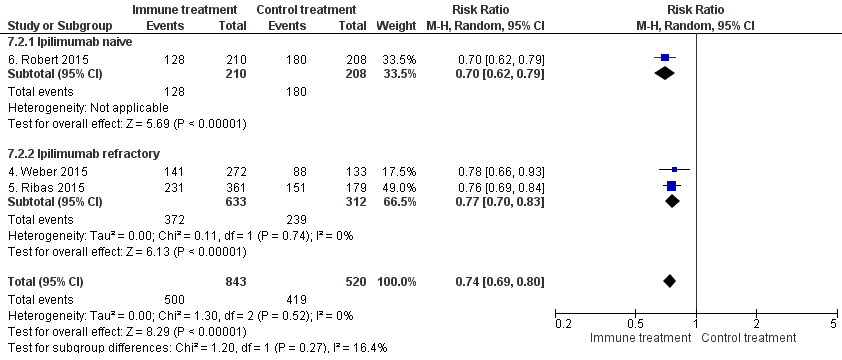
**

Forest plots of risk ratio for PFS from all available data. The size of the data markers (square) corresponds to the weight of the study in the meta-analysis. The effects of interventions are calculated with the random effects model. Criteria used for subgroup analyses include experimental agents (anti-CTLA-4 vs. anti-PD-1) (A) and response to prior ipilimumab treatment (ipilimumab naïve vs. refractory) in patient who were treated with anti-PD-1 treatment (nivolumab or pembrolizumab) (B).

**Supplementary Figure 4: Subgroup Analysis of 6 months ORR from Available Data**

**S4A: anti-CTLA-4 vs. anti-PD-1 treatment**

**
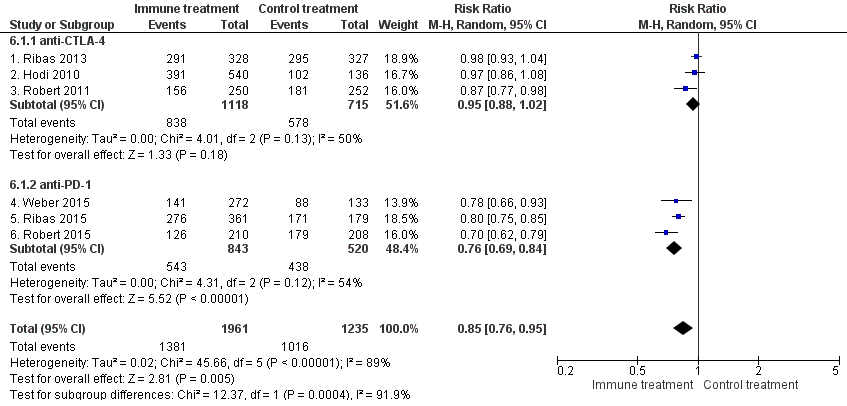
**

**S4B: ipilimumab naïve vs. ipilimumab refractory**

**
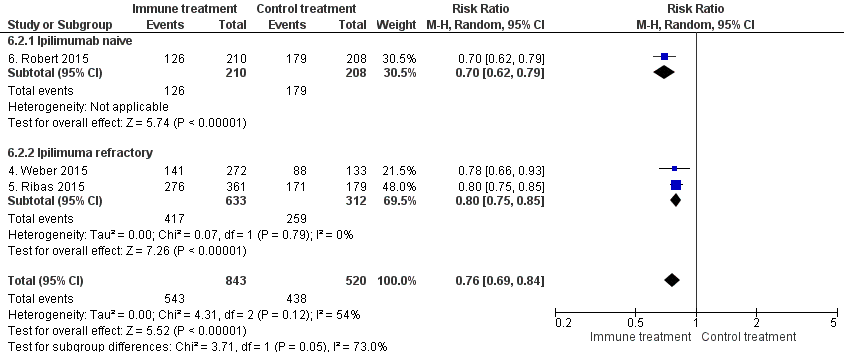
**

**S4C: BRAF wild type vs. mutant**

**
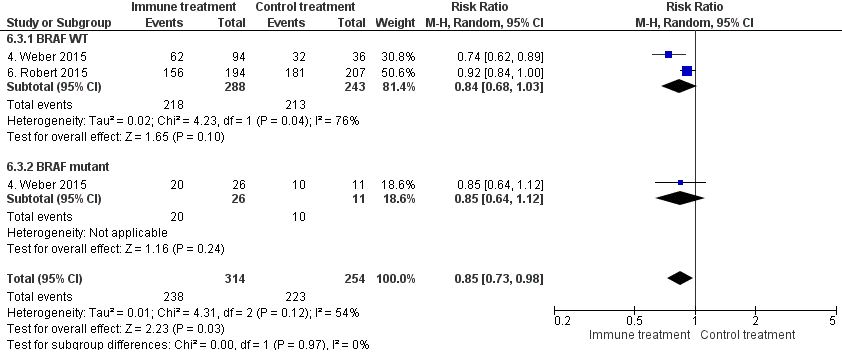
**

**S4D: PD-L1 positive vs. negative**

**
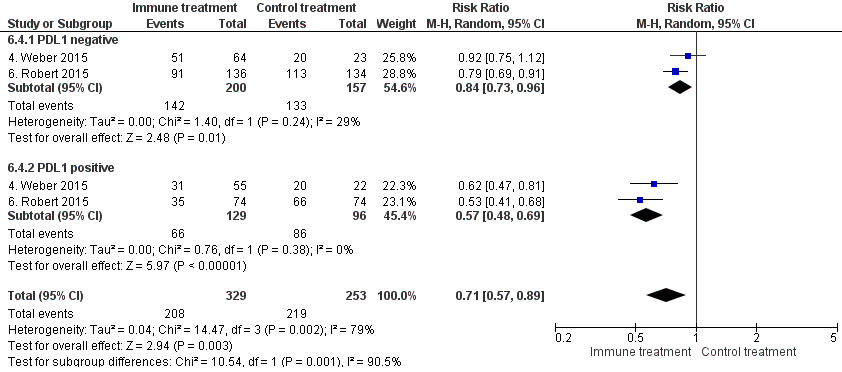
**

Forest plots of risk ration for ORR from all available data. The size of the data markers (square) corresponds to the weight of the study in the meta-analysis. Criteria used for subgroup analyses include experimental agents (anti-CTLA-4 vs. anti-PD-1) in all patients (A), response to prior ipilimumab treatment (ipilimumab naïve vs. refractory) (B), BRAF mutation status (wild type vs. mutation) (C), and PD-L1 positivity (at least vs. less than 5%) (D) in patients treated with anti-PD-1 treatments (nivolumab or pembrolizumab).

**Supplementary Table 1: Search Detail in PubMed, EMBASE and Cochrane Database**

| **Study** | **Search strategies** | **No. of Results** |
| --- | --- | --- |
| **PubMed** | (“Metastatic melanoma” [All Fields] OR “Cutaneous melanoma” [All Fields]) AND (“ipilimumab” [All Fields] OR “tremelimumab” [All Fields] OR “nivolumab” [All Fields] OR “pembrolizumab” [All Fields] OR “lambrolizumab” [All Fields] OR “CTLA-4” [All Fields] OR “PD-1” [All Fields]) AND (“controlled” [All Fields] OR “trial” [All Fields] OR “randomized” [All Fields]) AND ((“0001/01/01”[PDAT]: “2015/9/04”[PDAT]) AND “humans” [MeSH]) | 145 |
| **EMBASE** | 1. ‘Metastatic Melanoma’ OR ‘Cutaneous Melanoma’  2. AND (‘Ipilimumab’ OR ‘Tremelimumab’ OR ‘Nivolumab’ OR ‘Pembrolizumab’ OR ‘lambrolizumab’ OR ‘CTLA-4’ OR ‘PD-1’)/de  3. AND (‘Randomized Trial’)  3. AND (‘Human’)/de | 1. 38,021  2. 2,020  3. 305  4. 301 |
| **Cochrane**^¶^ | 1. ‘Metastatic Melanoma’ OR ‘Cutaneous Melanoma’  2. ‘Ipilimumab’ OR ‘Tremelimumab’ OR ‘Nivolumab’ OR ‘Pembrolizumab’ | 1. 929  2. 123 |

The initial literature search was completed on 2/2015 and updated on 9/2015.

^¶^ Cochrane Database of Systematic Reviews was used for the search of review articles.

**Supplementary Table 2: Additional Characteristics of Included Trials**

| **Study** | **Study period** | **Study design** | **Female (%)** | **PFS rate (%)** | | **Treatment Response rate (%)**^Ф^ | | **Grade 3/4 Adverse Events (%)** | | **Grade 3/4 Immune Related Adverse Events (%)** | | **Formula of Experimental Drug** |
| --- | --- | --- | --- | --- | --- | --- | --- | --- | --- | --- | --- | --- |
|  |  |  |  | **Exp** | **Ctrl** | **Exp** | **Ctrl** | **Exp** | **Ctrl** | **Exp** | **Ctrl** |  |
| **Hodi FS. 2010^1^**  **NCT00094653** | 2004-2008 | Multinational Multicenter  Double Blind  Randomized Controlled Phase III | 275 (41) | 14.4^¶^ | 5.1 | CR: 1 (0)  PR: 35 (6.5)  SD: 82 (15.2)  PD: 309 (57.2)  ND: 111 (20.6) | CR: 0 (0)  PR: 2 (1.5)  SD: 13 (9.6)  PD: 89 (65.4)  ND: 32 (23.5) | 233 (43.1)^†^ | 62 (45.6) | 58 (11.4) | 4 (3.0) | **Experimental group:** ipilimumab at 3mg/kg with and without gp100 peptide vaccine every 3 weeks, total 4 doses.  **Control group:** two modified HLA-A*0201 restricted peptides injected subcutaneously as an emulsion with incomplete Freund’s adjuvant. |
| **Robert C. 2011^2^**  **NCT00324155** | 2006-2008 | Multinational Multicenter  Double Blind Randomized Controlled Phase III | 201 (40) | 28.0 | 20.6 | CR: 4 (15.2)  PR: 34 (13.6)  SD: 45 (18.0)  PD: 111 (44.4)  ND: 56 (22.4) | CR: 2 (0.8)  PR: 24 (9.5)  SD: 50 (19.8)  PD: 131 (52.0)  ND: 45 (17.9) | 139 (55.6)^†^ | 69 (27.4) | 103 (41.7) | 15 (6.0) | **Experimental group:** ipilimumab at 10mg/kg every 3 weeks followed by dacarbazine 850mg/m2every 3 weeks through week 22.  **Control group:** placebo followed by dacarbazine 850mg/m2 every 3 weeks through week 22. |
| **Ribas A.**  **2013^3^**  **NCT00257205** | 2006-2007 | Multinational  Multicenter  Open Label  Randomized  Controlled  Phase III | 283 (43.2) | 94 | 95 | CR: 11 (3)  PR: 25 (8) | CR: 8 (2)  PR: 24 (7) | 170 (52) | 119 (37) | 74 (22.8) | 6 (1.9) | **Experimental group:** tremelimumab at 15mg/kg IV infusion once every 90 days for up to 4 cycles  **Control group:** dacarbazine at 1000mg/m2 IV on day 1 of a 21 day cycle or temozolomide at 200mg/m2 PO on days 1 to 5 of a 28 days cycle, up to 12 cycles or until disease progression, unacceptable toxicity or withdrawal of consent. |
| **Weber JS.**  **2015^4^**  **NCT01721746** | 2012-2014 | Multinational Multicenter  Open Label  Randomized Controlled Phase III | 144 (35.6) | 48 | 34 | CR: 4 (3.3)  PR: 34 (28.3)  SD: 28 (23.3)  PD: 42 (35)  ND: 12 (10) | CR: 0 (0)  PR: 5 (10.6)  SD: 16 (34.0)  PD: 15 (31.9)  ND: 11 (23.4) | 24 (9) ‡ | 32 (31) | 12 (5.0) | NR | **Experimental group:** nivolumab at 3mg/kg every 2 weeks, no dose reduction was allowed, but dose delays were allowed.  **Control group:** investigators’ choice of chemotherapy; either dacarbazine 1000 mg/m2 every 3 weeks, or carboplatin area under the curve 6 plus paclitaxel 175 mg/m2 every 3 weeks by IV infusion. Dose reduction was allowed. |
| **Robert C.**  **2015^5^**  **NCT01721772** | 2013-2014 | Multinational Multicenter  Double Blind Randomized Controlled Phase III | 172 (41.1) | 39.0 | 13.4 | CR: 16 (7.6)  PR: 68 (32.4)  SD: 35 (16.7)  PD: 69 (32.9)  ND: 22 (10.5) | CR: 0 (0)  PR: 5 (10.6)  SD: 16 (34.0)  PD: 15 (31.9)  ND: 32 (15.4) | 70 (34)‡ | 78 (38) | 12 (5.8) | 3 (1.5) | **Experimental group:** nivolumab at 3mg/kg every 2 weeks plus dacarbazine matched placebo every 3 weeks.  **Control group:** 1000mg/m2 of dacarbazine plus a nivolumab matched placebo every 2 weeks. |
| **Ribas A.**  **2015^6^**  **NCT01704287** | 2012-2013 | Multinational Multicenter  Double Blind  Randomized Controlled Phase II | 213 (39.4) | 34 (2 mg/kg)  38 (10 mg/kg) | 16 | CR: 9 (3)  PR: 75 (20.8)  SD: 63 (17.5)  PD: 170 (47.1)  ND: 44 (12.2) | CR: 0 (0)  PR: 8 (4)  SD: 33 (18)  PD: 111 (62)  ND: 27 (15) | 45 (13)^Ω^ | 47 (26) | 9 (<1) | 1 (<1) | **Experimental group:** pembrolizumab 2 or 10 mg/kg every 3 weeks  **Control group**: investigators’ choice of chemotherapy, either carboplatin + paclitaxel, carboplatin, paclitaxel, dacarbazine, or temozolomide. |

^†^ Adverse events were graded according to the National Cancer Institute’s Common Terminology Criteria for Adverse Events, version 3.0.

^¶^ PFS in this study was reported at 8 months. PFS in other studies were measured at 6 months.

^Ф^ Treatment response was assessed by Response Evaluation Criteria in Solid Tumors (RECIST) version 1.1-defined^7^.

^‡^ Graded adverse events according to the National Cancer Institute Common Terminology Criteria for Adverse Events, version 4.0.

^Ω^ This study reported grade 3-5 adverse events.

Abbreviation: PFS (progression free survival), NR (not reported), ND (could not be determined), CR (complete remission), PR (partial remission), SD (stable disease), PD (progression of disease).

**Supplementary Table 3: Risk of Bias Assessment of Studies According to Cochrane Risk Bias Assessment Tool ^8^**

| **Study** | **Random sequence generation** | **Allocation concealment** | **Blinding ^a^** | **Incomplete outcome data** | **Selective reporting** | **Other Source of Bias** |
| --- | --- | --- | --- | --- | --- | --- |
| **Hodi FS. 2010^1^**  **NCT00094653** | Adequate (permuted block) | Adequate (central allocation) | Unclear ^b^ | Adequate | Adequate | 1. Included only HLA-A*0201 positive patients. 2. No data reported regarding BRAF mutation and PD-L1 positivity. 3. Control group was treated with gp100, not chemotherapy. 4. PFS was reported at 8 months rather than 6 months. |
| **Robert C. 2011^2^**  **NCT00324155** | Adequate (minimization) | Adequate (central allocation) | Adequate | Adequate | Adequate | 1. 10mg/kg of ipilimumab dose was used in this study. 2. Experimental group was treated with ipilimumab and dacarbazine. 3. No data reported regarding BRAF mutation and PD-L1 positivity. 4. 74% of patients were treatment naïve. |
| **Ribas A.**  **2013^3^**  **NCT00257205** | Adequate (minimization) | Adequate (central allocation) | Unclear ^c^ | Adequate | Adequate | 1. Included only treatment naïve patients. 2. Choice of chemotherapeutic agent was at the discretion of the investigator. 3. 16% of patients in the control arm received ipilimumab treatment as salvage regimen. |
| **Weber JS.**  **2015^4^**  **NCT01721746** | Adequate (permuted block, automated interactive voice response system) | Adequate (central allocation) | Adequate | Adequate | Adequate | 1. Patients with BRAF ^WT^ must have had progression after anti-CTLA-4 treatment. 2. Patients with BRAF^V600E^ mutation must have had progression on anti-CTLA-4 treatment and a BRAF inhibitor. 3. Exclude patients with previous anti-PD-1, anti-PD-L1 or anti-PD-L2 antibodies. 4. Excluded patients who had grade 4 toxic effects or used infliximab to manage adverse events from ipilimumab treatment. 5. 17% of patients randomized to ICC withdrew consent compared with only 1 patient (<1%) in the nivolumab group. 6. Treatment response of 23.4% patients in ICC group cannot be assessed. |
| **Robert C.**  **2015^5^**  **NCT01721772** | Adequate (automated interactive voice response system) | Adequate (central allocation) | Adequate | Adequate | Adequate | 1. Included previously untreated patients without BRAF mutation. |
| **Ribas A.**  **2015^6^**  **NCT01704287** | Adequate (centralized interactive voice response system) | Adequate (central allocation) | Adequate | Adequate | Adequate | 1. Only included patients with progressive disease after ipilimumab treatment, if BRAF^V600^ positive, previous treatment with BRAF or MEK inhibitor or both. 2. 48% of patients in the control arm who had PD were crossed over to pembrolizumab treatment. 3. No data reported regarding PD-L1 positivity. 4. Open label for the treatment assignment of pembrolizumab vs. chemotherapy, but double blinded for the assignment of the dose of pembrolizumab dose. |

^a^ The adequacy of blinding was judged by the blindness of outcome assessment since the progression free survival and overall response rate are the primary outcome measures.

^b^ Treatment response assessment was performed at the study sites and no central reading was obtained.

^c^ Tumor data assessed by investigators were reviewed by the sponsor to ensure compliance with RECIST criteria.

**Reference**

1 Hodi, F. S. *et al.* Improved survival with ipilimumab in patients with metastatic melanoma. *N Engl J Med* **363**, 711-723, doi:10.1056/NEJMoa1003466 (2010).

2 Robert, C. *et al.* Ipilimumab plus dacarbazine for previously untreated metastatic melanoma. *N Engl J Med* **364**, 2517-2526, doi:10.1056/NEJMoa1104621 (2011).

3 Ribas, A. *et al.* Phase III randomized clinical trial comparing tremelimumab with standard-of-care chemotherapy in patients with advanced melanoma. *J Clin Oncol* **31**, 616-622, doi:10.1200/JCO.2012.44.6112 (2013).

4 Weber, J. S. *et al.* Nivolumab versus chemotherapy in patients with advanced melanoma who progressed after anti-CTLA-4 treatment (CheckMate 037): a randomised, controlled, open-label, phase 3 trial. *Lancet Oncol* **16**, 375-384, doi:10.1016/S1470-2045(15)70076-8 (2015).

5 Robert, C. *et al.* Nivolumab in previously untreated melanoma without BRAF mutation. *N Engl J Med* **372**, 320-330, doi:10.1056/NEJMoa1412082 (2015).

6 Ribas, A. *et al.* Pembrolizumab versus investigator-choice chemotherapy for ipilimumab-refractory melanoma (KEYNOTE-002): a randomised, controlled, phase 2 trial. *Lancet Oncol* **16**, 908-918, doi:10.1016/S1470-2045(15)00083-2 (2015).

7 Eisenhauer, E. A. *et al.* New response evaluation criteria in solid tumours: revised RECIST guideline (version 1.1). *European journal of cancer (Oxford, England : 1990)* **45**, 228-247, doi:10.1016/j.ejca.2008.10.026 (2009).

8 Moher, D., Liberati, A., Tetzlaff, J., Altman, D. G. & Group, P. Preferred reporting items for systematic reviews and meta-analyses: the PRISMA statement. *J Clin Epidemiol* **62**, 1006-1012, doi:10.1016/j.jclinepi.2009.06.005 (2009).
